# Ensemble CNN for colon cancer detection using histopathological image

**DOI:** 10.3389/fonc.2026.1674606

**Published:** 2026-04-27

**Authors:** Akshay Kulkarni, Amit Upadhyay, Monika Mangla, Swati Aggarwal

**Affiliations:** 1 Department of Information Technology, Dwarkadas J. Sanghvi College of Engineering, Mumbai, India; 2 Department of Logistics, Molde University College, Molde, Norway

**Keywords:** colon cancer, deep learning, ensemble model, histopathology, image preprocessing

## Abstract

Colon cancer remains one of the leading causes of cancer-related mortality worldwide, with delayed detection significantly impacting survival rates. The time-consuming nature of traditional histopathological analysis contributes to these diagnostic delays. This study leverages advances in artificial intelligence to improve both the speed and accuracy of colon cancer detection through automated analysis of tissue sample images. We propose a weighted ensemble model comprising four convolutional neural networks namely RegNet X, RegNet Y, Swin B, and VGG11, that enhances prediction accuracy by combining outputs from base models. The prime focus of the research is to devise a recall prioritized ensemble model capable of detecting various types of colon cancers, while also examining the possibility of the model to generalize beyond the training domain. The ensemble was trained using a dataset of 10,000 histopathological images of colon tissues containing both benign and malignant samples. We introduce a novel preprocessing pipeline incorporating histogram equalization and contrast stretching for image enhancement with a motive to improve diagnostic accuracy. Notably, the ensemble model demonstrated superior recall metrics compared to traditional ML approaches—a critical measurement in healthcare applications. The ensemble model proposed has a test accuracy of 99% and a recall of almost 100%. Further, the proposed model is also tested for lung cancer to validate its generalizability. The experimental results obtained establish the validation of proposed model in terms of generalizability, demonstrating strong performance.

## 1 Introduction

Cancer has been a significant global health concern for decades, with high mortality rates often attributed to delayed detection. Despite advancements in diagnostic methods that have improved medical care, the challenge of early and accurate detection still prevails (1). This is particularly critical for colon cancer, a major health issue caused by the uncontrolled growth of cells in the large intestine and rectum. Colon cancer often originates from resectable polyps, highlighting the importance of early diagnosis. Traditional diagnostic methods such as colonoscopy and histopathology, although effective, are time-intensive, costly, and reliant on highly skilled pathologists (2). These diagnostic challenges can delay treatment initiation, potentially leading to fatal outcomes. Consequently, there is an urgent need for efficient and automated diagnostic systems capable of timely and accurate disease detection.

This study proposes an ensemble model based on Convolutional Neural Networks (CNNs) to address these challenges. CNNs have demonstrated remarkable success in image classification across various domains, including healthcare, agriculture, and education (3). Leveraging this capability, the proposed system processes histopathological images through an ensemble of four CNN models—RegNet X, RegNet Y, Swin B, and VGG11—to efficiently diagnose colon cancer. The primary goal of this research is to enhance recall rates alongside accuracy, ensuring that no cancerous cases are overlooked. This focus on recall is particularly significant in healthcare, as misclassified positive cases can cause delayed treatment (4).

The motivation for prioritizing recall stems from alarming statistics reported by GLOBOCAN (Global Cancer Statistics), which highlight the prevalence of misclassified positive cases in cancer detection (5). By improving recall and accuracy, the proposed model aims to address this critical issue. Furthermore, advanced image preprocessing techniques such as histogram equalization and contrast stretching are employed to mitigate challenges such as uneven staining protocols and scanner hardware variations that can obscure pathological features in histopathological images (6) (7).

In addition to enhancing recall, the proposed model also aims to develop a generalizable system, efficient in handling other kind of cancers too. In order to validate the generalizability of proposed model, its performance is tested on lung cancer dataset despite its prime focus on colon cancer diagnosis. This dual focus of recall enhancement and generalizability aims to establish the model’s potential for broader application across different types of cancers.

The objectives of the current research are:

1. To improve recall rates in cancer diagnosis, ensuring no positive cases are misclassified.
2. To examine the possibility of developing a generalizable model capable of detecting multiple types of cancer.

In order to achieve these objectives, current research work is organized into various sections. Here, section 1 discusses the motive for research and various research objectives. Related work carried out by researchers is presented in section 2. Proposed model is discussed in section 3. Results are discussed in section 4. Finally, conclusion and future work is given in section 5.

## 2 Literature review

This section examines significant contributions by researchers in the field of automated colon cancer detection using deep learning techniques. Among various types of cancers, colorectal cancer (CRC) remains one of the most prevalent malignancies globally, accounting for approximately 10% of all cancer-related deaths. With an estimated 1.9 million new cases diagnosed annually, colon cancer—a subset of CRC alongside rectum cancer—represents a major healthcare concern. Like several other types of cancers, CRC also necessitates early diagnosis as localized tumors exhibit a 90% survival rate compared to just 14% for metastatic cases (1). However, traditional methods involving manual examination of biopsy slides, is labor-intensive, time-consuming, and susceptible to human error, particularly when distinguishing benign adenomas from early-stage adenocarcinomas, posing significant challenges (8, 9). These challenges involved in manual examination of the disease highlight the limitations establishing the imperative need for automated diagnostic systems. Resultantly, several researchers have attempted to propose automated system for CRC diagnosis utilizing the recent advancements in the technology.

The most notable contribution in this direction was carried out by authors in (10) who created a high-quality image dataset comprising of 25,000 histopathological 768×768 pixel images spanning across five different classes namely Colon adenocarcinoma, Benign colonic tissue, Lung adenocarcinoma, Lung squamous cell carcinoma, and Benign lung tissue. This newly created balanced dataset enabled future researchers to carry out equitable training although this research work itself did not provide any ML model. Building upon multi-class histopathological image datasets, authors in Uddin et al. (11) proposed robust and efficient neural network models for simultaneous classification of colon and lung cancer from multi-modal images, thus setting a baseline for comparison of ensemble models. During research, the prime challenge observed by various researchers is complex background and small size of the object. This challenge was addressed by exploiting the competence of deep learning owing to its exceptional capability in extracting spatial features from histopathological images. For instance, authors in (12) suggested an improved faster R-CNN with multi-loss function and multi-scale detection. Incorporation of multi scale detection in (12) not only retains the semantic information but also captures the detail about edges of the image. During the experimental evaluation, it was observed that improved Faster R-CNN outperforms Faster R-CNN by 2.4%. Furthermore, it was observed by authors in Li et al. (13) that performance improvement is largely influenced by hyperparameter optimization. They optimized learning rate, batch size, and optimizer settings using the Multi-Strategy Parrot Optimizer (MSPO) approach, which led to improved robustness and increased classification accuracy. Similarly authors in Nabeel et al. (14) utilized hyperparameter tuning, focusing on the Gamma and C parameters, while achieving an accuracy of 99.16%. Authors in Yang et al. (15) used a dynamic Snow Leopard Optimization algorithm to optimize key hyperparameters of an Inception-V4 network to improve recall and detection performance for diabetic retinopathy, demonstrating effectiveness of optimization-based pipelines in improving sensitivity-oriented medical imaging models.

The employment of deep learning was also validated by authors in (16) by using different pre-trained networks. The proposed framework achieved an impressive 0.97 AUC (Area Under the Curve) and 96.98% accuracy using RegNet models for patch-level classification (16). Although, research yields promising results, concerning aspect is computationally intense nature of the framework, limiting of proposed framework. This computationally intensive concern of framework proposed in (16) is addressed by researchers in (17) by proposing a lightweight CNN MA ColonNET optimized specifically for adenocarcinoma classification. The proposed framework consisted of 45 layers and achieved an accuracy of 99.75%. Motivated by the promising results obtained by researchers, authors in (18) proposed a CNN architecture incorporating enhanced convolutional learning modules (ECLMs), attention learning module (ALM), and transitional modules (TMs). The proposed model resulted in focused and enhanced feature extraction escalating the efficiency of CRC diagnosis by achieving 98.2% accuracy and 97.8% F1-score. Authors in (19) investigated the efficiency of various DL architectures, including ResNet50, EfficientNetB0, ViT, and MobileViT in addition to different stain normalization methods and image-denoising techniques. Authors also proposed an ensemble model by combining a CNN and a transformer enabling it to fetch intricate local patterns and relationships among regions spanning across image. The proposed ensemble model is capable of achieving 92.02% accuracy. Continuing the research further, authors in Yang et al. (20) extracted gray-level co-occurrence matrix (GLCM) texture features from lung MRI scans and employed intelligent Bayesian methods with posterior probability estimation, achieving reliable lung cancer prediction and establishing a probabilistic baseline that supports cross-classification and ensemble-based diagnostic frameworks.

While numerous researchers have attempted to provide efficient framework for disease diagnosis, authors in (8) highlighted the inability to utilize full potential of deep learning due to intricacies involved in processing histopathological images such as outlier detection. Authors also raised the concern regarding lack of interpretability, causing a major bottleneck. Authors in (8) also underlined the need of incorporating explainability in the disease diagnosis, opening avenues for employing explainable artificial intelligence (XAI). This requirement of XAI put forth by authors in (8) was addressed by authors in (21) by proposing a CNN model to classify and predict two different types of cancer using histopathological images. Additionally, authors also attempted XAI techniques to enhance the interpretability of the model.

Systematic review on color correction and contrast enhancement methods conducted by authors in Lai et al. (22) reveals the effectiveness of histogram equalization and contrast stretching in improving the visibility of features, thus validating the use of these methods as strong preprocessing tools. In another study by authors in Xu et al. (23), a multi-task hybrid fusion-based enhancement method is proposed, emphasizing the fact that effective enhancement tools can significantly improve the performance of learning models in adverse visual conditions. Apart from challenge of handling complex and small images, automated disease detection also suffers from scarcity of the annotated datasets. In order to mitigate the data scarcity, authors in (24) proposed combining ResNet50 CNN and transfer learning. The proposed model was validated using experimental evaluation and it was observed that suggested model achieves 99.99% and 99.77% accuracy during training and testing respectively. Employment of transfer learning also enables authors in (24) to achieve reduction in training time by 40%. Further authors in (25) suggested employing a dialated ResNet and attention module for disease diagnosis and classification. The suggested framework achieves an accuracy of 98.75–99.76% on colorectal cancer histology datasets (25).

The requirement of data privacy and sensitivity is also a matter of paramount concern for healthcare data, necessitating the development of privacy-sensitive frameworks. In this direction, authors in (26) proposed a federated learning approach for disease diagnosis. Employment of federated learning ensures that data is trained locally without transmitting to a central location, a step toward achieving data privacy. Authors compared FedDropoutAvg with other Federated learning benchmark models. The performance achieved by employing federated learning is also promising and thus advocate its employment in disease diagnosis.

Building on this work, researchers in (9) fused features from EfficientNet-B7 and DenseNet-201, employing metaheuristic optimization techniques. The proposed model outperformed the state-of-the-art models by enhancing the tumor detection precision by 12%. Few other contributions carried out by other researchers evaluated on the LC25000 dataset have been illustrated in **Table 1** to provide a comprehensive comparison of various models for colon cancer detection. The comparison highlights model architectures, primary methodological contributions, and classification accuracy.

**Table 1:** Comparative analysis of various models in terms of accuracy.

Cite	Model	Dataset	Main achievement	Accuracy
Sethy et al. (27)	Hybrid (AlexNet, SVM)	LC25000	Hybrid network	99.30%
Hadiyoso et al. (28)	VGG16 swith CLAHE	LC25000	Image enhancement	98.96%
Wahid et al. (29)	Various CNNs	LC25000	Multiple models	99.87%
Iqbal et al. (30)	ColonNet	LC25000	High F1-score	96.31%
AlGhamdi et al. (31)	ShuffleNet, DCRNN	LC25000	Transfer learning	98.99%
Stephen et al. (32)	Neural Architecture Search	LC25000	Neural net search	93.91%
Kumar et al. (33)	Transfer Learning	LC25000	Feature extraction	98.60%

From the detailed survey of the related work, the efficacy of CNN models toward disease diagnosis is validated and advocated. It is worth noting that although numerous researchers have aimed to maximize the accuracy, recall has been depriving required attention despite its significance in healthcare domain. Authors in current research strongly believe that recall should also be considered as a significant measure, particularly for cancer diagnosis as a misclassified positive case may lead to fatality. Additionally, generalization in the cancer diagnosis has also taken a backseat in the related work. Authors in current work aim to propose a model with an objective to maximize recall in addition to accuracy while also analyzing the model’s capability to perform on unseen data distributions.

## 3 Methodology

**Figure 1:**
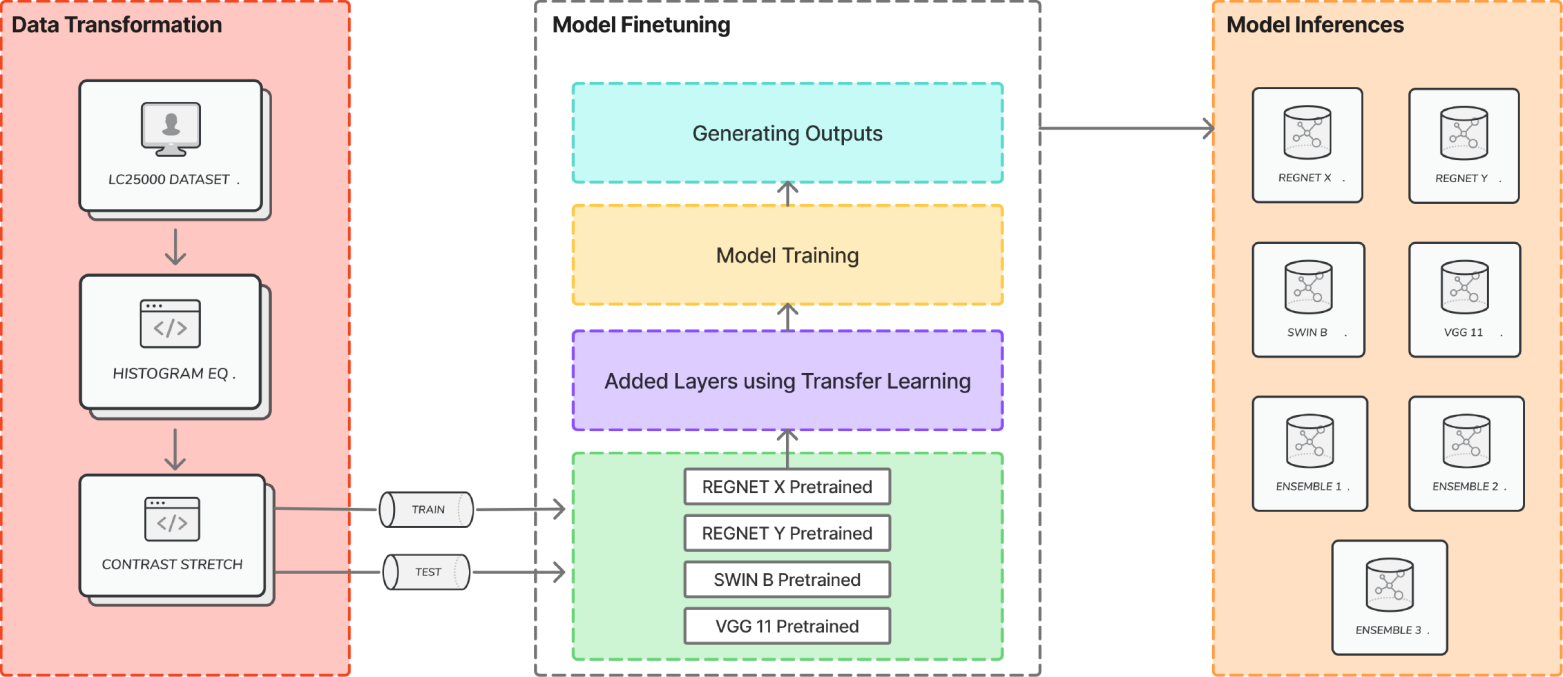
Illustration of the proposed architecture.

The proposed methodology integrates advanced DL models, transfer learning, and ensemble modeling to enhance the efficiency for disease diagnosis and identification. The proposed methodology comprises various phases namely data transformation, model fine-tuning, and ensemble modeling as illustrated in **Figure 1**.

### 3.1 Data transformation

This is the first step in the proposed model which primarily aims toward data collection, data preparation through various preprocessing techniques and finally splitting of data in training, testing and validation data. Thus, the various steps in data transformation are following:

#### 3.1.1 Dataset used

**Figure 2:**
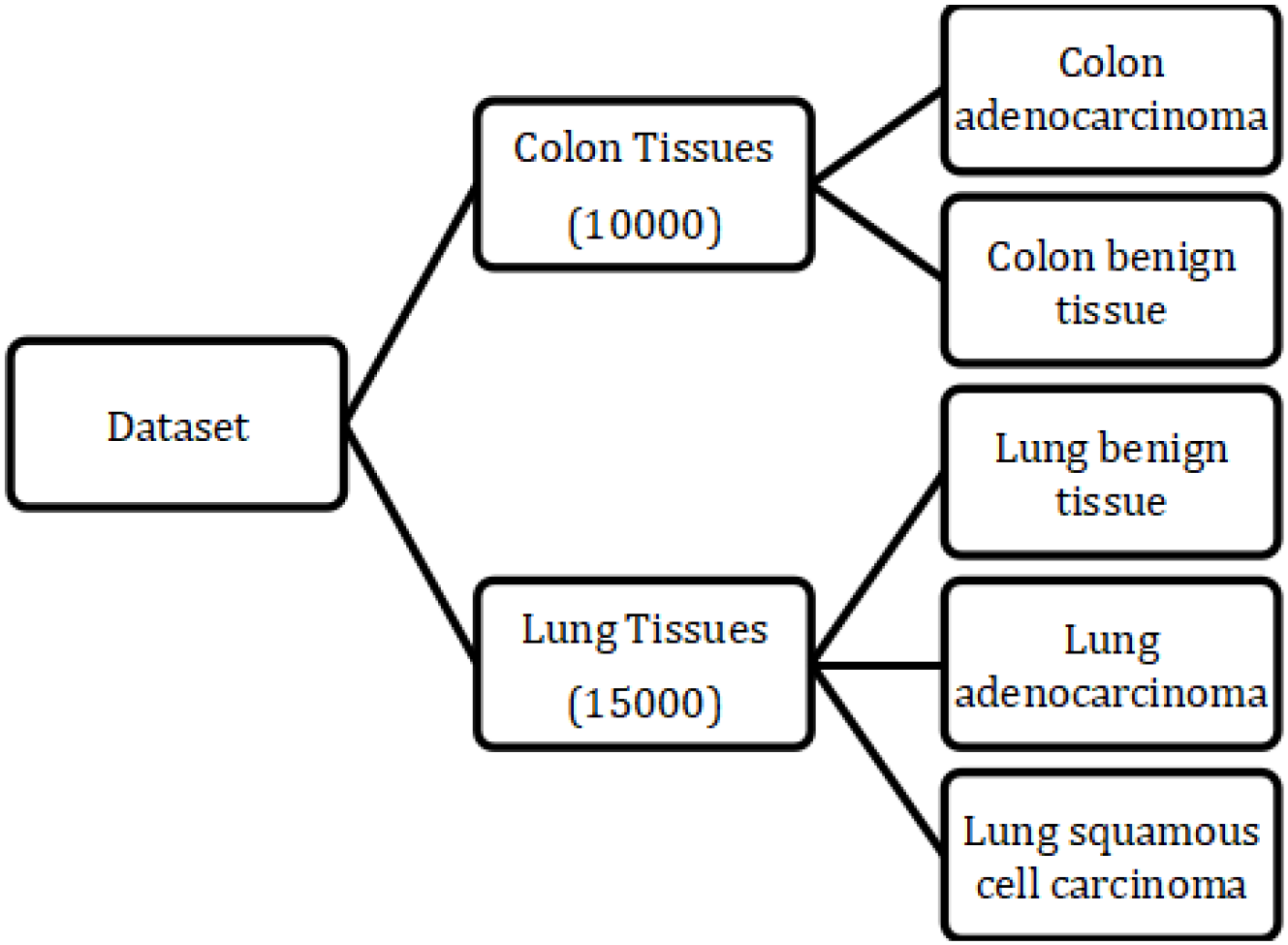
Illustration of the dataset.

The first and foremost step of the proposed methodology is selection of dataset. For current study, authors have utilized a comprehensive dataset containing 25,000 histopathological images, derived from HIPAA compliant and validated sources, ensuring that all images are handled with the utmost regard for patient privacy and data integrity (10). This dataset comprises of 15000 images of lung cancer tissues, generated from an original sample of 750 images of lung tissue (250 images each of benign lung tissue, lung adenocarcinomas, and lung squamous cell carcinomas). Additionally, 10,000 colon cancer images are generated from original sample of 500 images of colon tissue (250 each of Colon adenocarcinoma and Colon benign tissue) further expanding the variety of cases. The hierarchical structure of the dataset is illustrated in **Figure 2**.

The considered dataset illustrated in **Figure 2** enables targeted analysis and thus enhances the efficiency of proposed model to discern among different types of tissues and associated pathologies.

#### 3.1.2 Data sampling

In proposed model, data sampling has a vital contribution in ensuring that the training process is both efficient and effective. For the same, authors employed Fisher-Yates shuffle technique to randomly shuffle the dataset ahead of its splitting into training batches (34). Employment of the Fisher-Yates shuffle technique guarantees that each permutation of the array is equally likely, which is crucial for achieving a balanced and representative sampling of the data. Shuffling of the images ensures that each batch contains a diverse mix from all different classes of images. Since there are no patient-level identifiers in the LC25000 dataset, subject-wise data partitioning is not possible. While the Fisher-Yates is a widely used method, it may lead to some risk of leakage due to image-level correlations, especially without data augmentation. This could not be further verified in the case of the colon dataset. Yet, for lung cancer predictions, there were no signs of data leakage, and the model performed stably without any additional training.

#### 3.1.3 Data preprocessing

In the proposed methodology, considered histopathological images are preprocessed ahead of training. Histogram equalization and contrast stretching are employed to enhance the quality of histopathological images with a motive to improve diagnostic accuracy. Histogram equalization redistributes pixel intensities across the image, increasing global contrast and making critical features more distinguishable. On the other hand, contrast stretching adjusts pixel intensity values to span across the entire dynamic range, enhancing the visibility of subtle details in the image. These techniques complement each other by addressing different aspects of contrast enhancement: histogram equalization focuses on redistributing intensity values based on their frequency, while contrast stretching applies a linear transformation to expand the intensity range.

The preprocessing pipeline is engineered to enhance image quality without compromising diagnostically important color features. Histogram equalization is applied selectively to the Y channel in the YCrCb color space since Y channel contains brightness information. This enables achieving brighter and clearer image without warping chromatic data important for histopathological interpretation. To further normalize image intensity distributions, contrast stretching is adopted to rescale pixel values to the range of [0–255], correcting for scanner or staining-induced variation among samples.

During this preprocessing, one crucial step is normalizing pixel values from a range of 0–255 to 0-1. This normalization helps to stabilize the training process by ensuring that the input values are on a consistent scale, leading to faster convergence during optimization.

**Figure 3:**
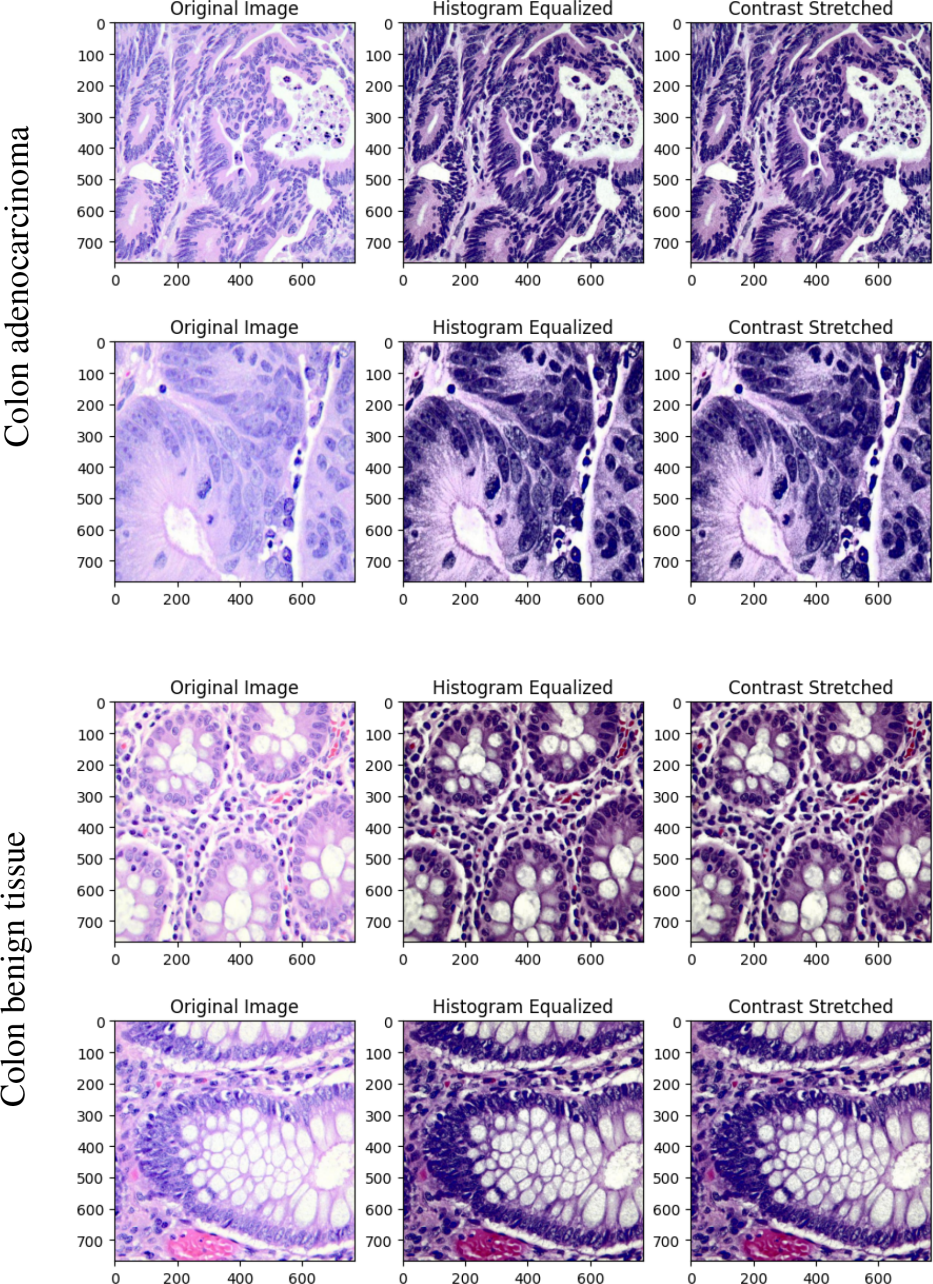
Original, histogram equalized and contrast stretched images of colon tissue.

Enhancement of image contrast and feature visibility enables CNNs to extract features more effectively during training and inference. The impact of these preprocessing methods is illustrated in **Figure 3**, that is indicative of advanced image preprocessing in developing robust automated systems for disease detection.

### 3.2 Model selection

The proposed ensemble model integrates four CNN architectures—RegNet X, RegNet Y, Swin B, and VGG11—each known for its complementary strengths in tackling the intricacies involved in histopathological image analysis. This approach reflects advancements in ensemble-based deep learning, which consistently outperform single-model architectures in cancer detection tasks.

RegNet X and RegNet Y form the foundation of efficiency and robustness within the ensemble. RegNet X, with 54.3 million parameters, balances computational efficiency and accuracy through regularization strategies, making it well-suited for generalizable feature extraction. RegNet Y, comprising 83.6 million parameters, builds on this by introducing flexible depth and width adjustments to capture intricate patterns in malignant tissues. Both models effectively address overfitting risks that often arise from complex imaging data.

Swin B Transformer, with 87.8 million parameters, uses a hierarchical self-attention mechanism that excels at identifying long-range dependencies and subtle morphological variations, such as irregular glandular structures or nuclear atypia. While its high capacity enhances sensitivity to fine details, it also increases susceptibility to overfitting—a challenge mitigated by integrating stabilizing architectures.

VGG11, containing 138.4 million parameters, complements Swin B’s complexity with its straightforward sequential convolutional layers and integrated self-attention modules. Known for its reliability in feature extraction, VGG11 enhances both local and global contextual understanding while reducing overfitting through its simplified architecture.

The ensemble model leverages the distinct strengths of these architectures through several key strategies:

1. Complementary Feature Extraction: RegNet X/Y focus on efficient pattern recognition, while Swin B and VGG11 specialize in capturing spatial hierarchies and stabilizing predictions.
2. Overfitting Mitigation: Combining diverse architectures minimizes reliance on any single model’s biases, ensuring more consistent diagnostic results.
3. Aggregation Strategy: Predictions are fused using techniques such as weighted averaging to harmonize outputs from the different models and reduce variance.

The proposed architecture adopts multi-model approach to effectively address challenges inherent in histopathological analysis, such as staining variability and scanner artifacts. By distributing feature learning across base models with varying inductive biases, it is expected that the ensemble achieves a robust framework that enhances diagnostic accuracy while maintaining generalizability. This is particularly critical for clinical applications where minimizing false negatives is essential for timely treatment and improved patient outcomes.

#### 3.2.1 RegNet X

**Figure 4:**
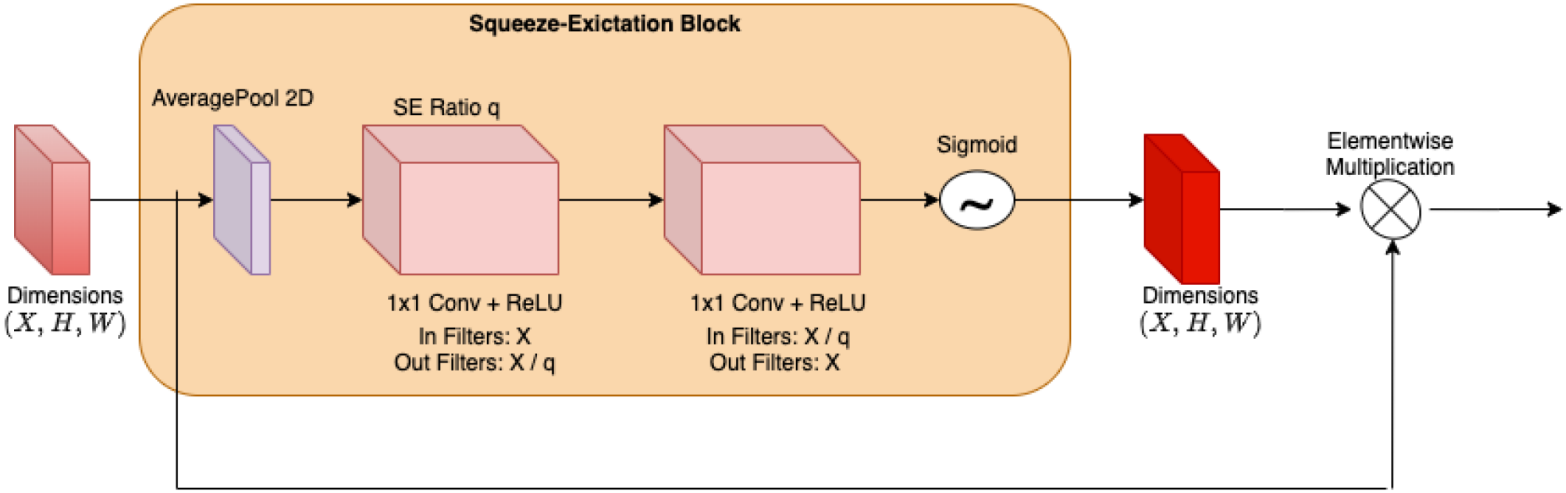
Architecture of RegNet X model.

This model is designed to optimize the efficiency through a regularization strategy that enhances generalization. Its architecture as illustrated in **Figure 4** is tailored to provide a balance between performance and computational efficiency, making it suitable for current task.

The core of a residual block is defined as:


$$y=F(x,\{W_{\text{i}}\})+x$$

Where:

• x is the input to the residual block.

• $F(x,\{W_{i}\})$ represents the residual function, typically consisting of convolutional layers with weights $W_{i}$.

• *y* is the output of the residual block.

This formulation allows the network to learn residual mappings, which simplifies the learning process and mitigates the problem of vanishing gradients.

#### 3.2.2 RegNet Y

**Figure 5:**
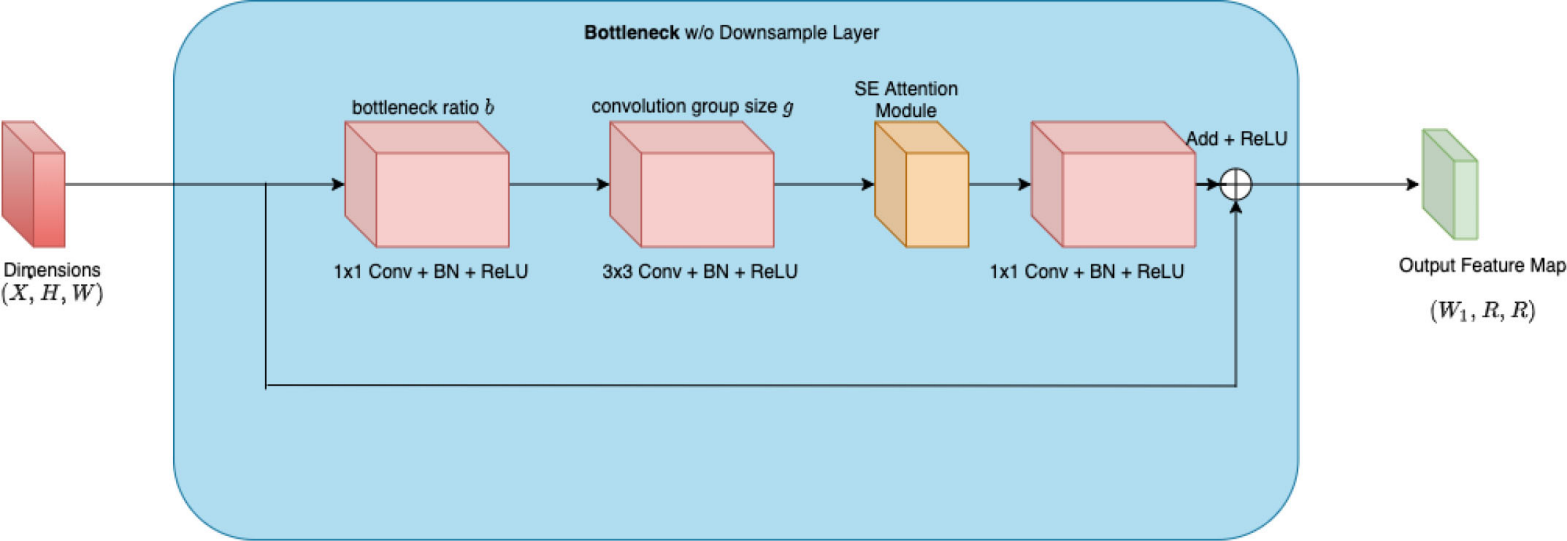
Architecture of RegNet Y model.

RegNet Y builds upon the strengths of its predecessor by introducing additional flexibility in its design. This model as illustrated in **Figure 5** is particularly adept at learning complex patterns in data, which is crucial in distinguishing between benign and malignant tissues.

The core of a residual block is defined as:


$$y=F(x,\{W_{i}\})+x$$

Where:

• x is the input to the residual block.

• $F(x,\{W_{i}\})$ represents the residual function, typically consisting of convolutional layers with weights $W_{i}$.

• *y* is the output of the residual block.

This formulation allows the network to learn residual mappings, which simplifies the learning process and mitigates the vanishing gradient problem.

#### 3.2.3 Swin B

**Figure 6:**
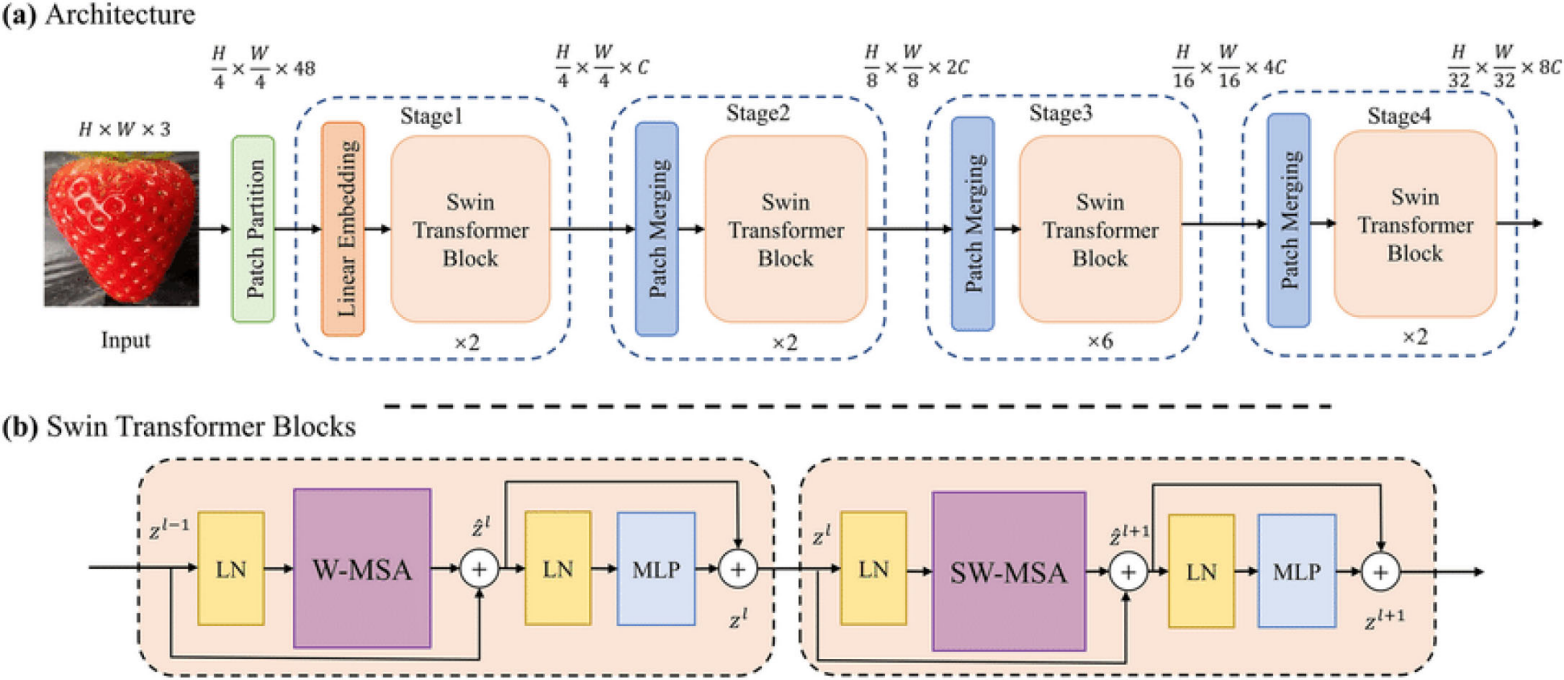
Architecture of Swin B model.

The Swin B model utilizes a unique hierarchical architecture that processes images at various scales as illustrated in **Figure 6**.

The capability of Swin B allows it to capture intricate details in the histopathological images, which can be vital for accurate classification. The output *y* of a Swin Transformer block is computed as:


y=M(x)+x

Where:

• x is the input to the block.

• M(x) represents the multi-head self-attention operation within the window.

• *y* is the output of the block.

This formulation allows the model to capture both local and global dependencies efficiently.

#### 3.2.4 VGG11

**Figure 7:**
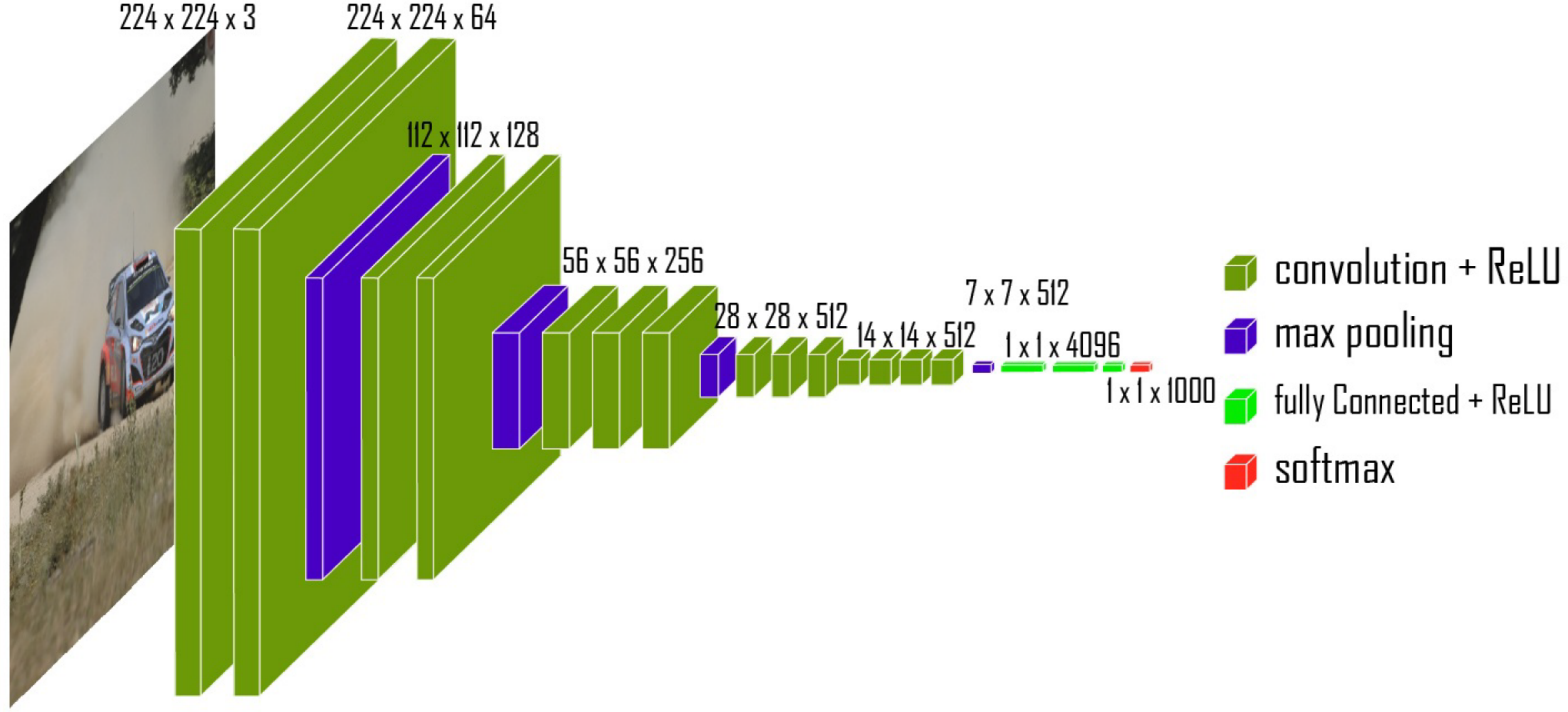
Architecture of VGG 11 model.

This well-established model is recognized for its depth and comprehensive feature extraction capabilities. VGG11’s architecture as illustrated in **Figure 7** is particularly effective in recognizing fine-grained details, which is essential when analyzing histopathological images.

The output *y* of the network is computed as:


$$y=W_{fc}\cdot (\sigma (W_{\text{conv}}\cdot x+b_{\text{conv}}))+b_{fc}$$

Where:

• x is the input image.

• $W_{\text{conv}}$ and $b_{\text{conv}}$ are the weights and biases of the convolutional layers.

• σ denotes the activation function (e.g., ReLU).

• $W_{fc}$ and $b_{fc}$ are the weights and biases of the fully connected layers.

• *y* is the final output of the network.

### 3.3 Ensemble model

**Figure 8:**
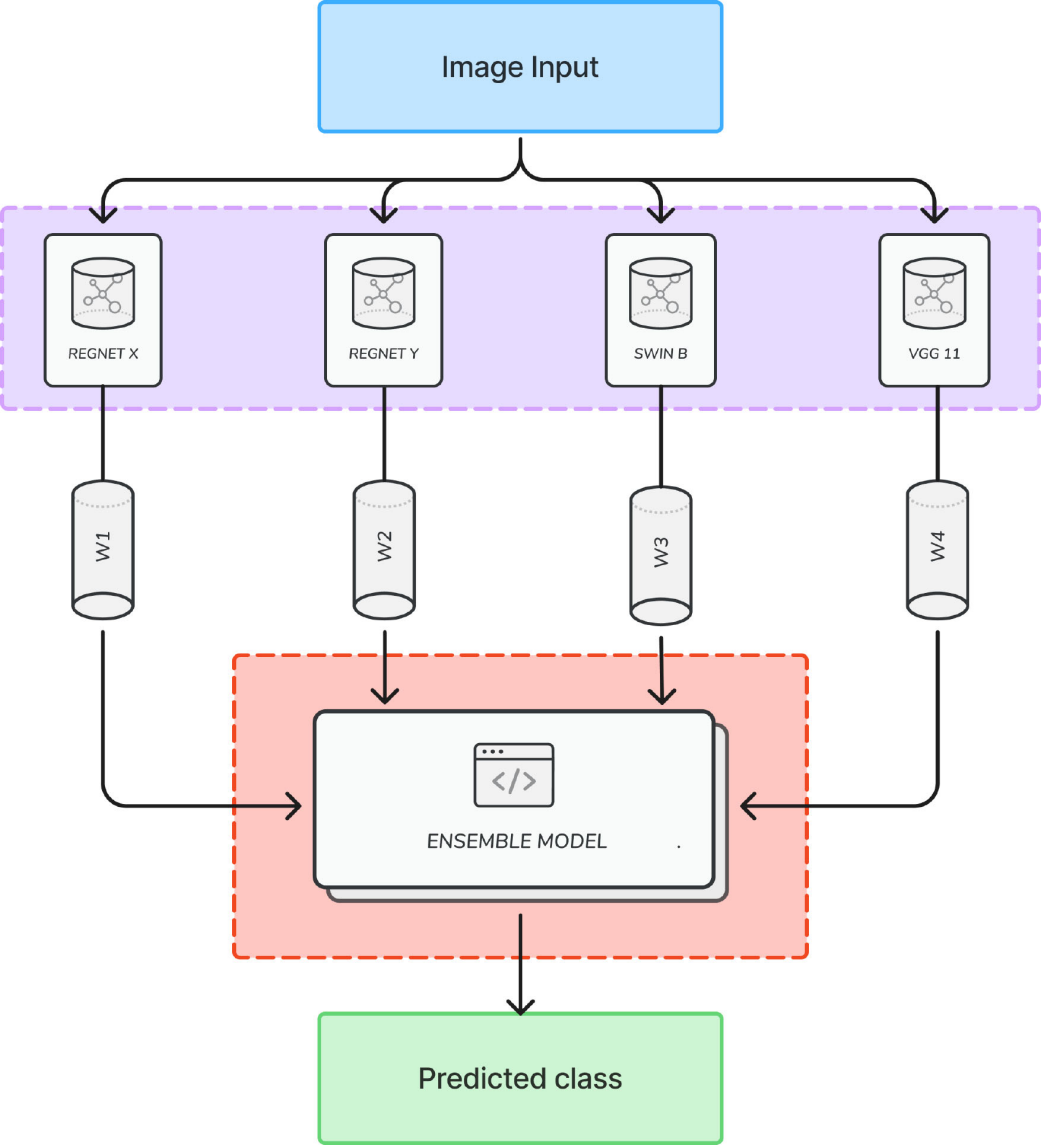
Illustration of proposed ensemble model.

Now, as discussed earlier, the rationale behind employing an ensemble methodology for cancer detection lies in the inherent complexity of histopathological images. Each individual CNN possesses unique strengths and weaknesses which may affect its performance for specific instance of the dataset. By combining the predictions from multiple models, collective intelligence can be leveraged to enhance overall classification accuracy and reliability, as illustrated in **Figure 8**.

The base models, pretrained on the IMAGENET1K V1 dataset, effectively extract relevant features from histopathological images. The diversity of these architectures mitigates overfitting risks and improves generalization to unseen data, critical in medical applications where misclassifications can have serious consequences. The ensemble methodology also fosters consensus among models, increasing confidence in predictions. This is particularly beneficial for detecting subtle differences between benign and malignant tissues, where minor variations can significantly influence diagnostic outcomes.

To optimize performance, the proposed ensemble employs a weighted averaging strategy for combining outputs from the base models as illustrated in **Figure 8**. Weights are assigned dynamically based on the performance of base models during experimental evaluations, with higher weights given to models that outperform others. This adaptive weighting ensures that the ensemble maximizes predictive accuracy while maintaining robustness. By harnessing the collective strengths of diverse CNN architectures, this approach aims to achieve reliable and accurate classification of histopathological images, contributing to improved cancer detection methodologies. The complete code for proposed methodology can be found at https://github.com/AkshayKulkarni3467/Ensemble-Model-For-Colon-Cancer.

## 4 Results and discussion

This section discusses the results during experimental evaluation of the proposed model.

### 4.1 Experimental setup

The experiment is carried out on a system equipped with an NVIDIA GeForce GTX 1650 Graphical Processing Unit (GPU) (4GB VRAM) providing necessary computational resources for training deep learning models. Each model is trained for 50 epochs with a train-test split of 80:20 on the 10,000 colon cancer images with random seed 45010. Authors have considered a batch size of 32 images for each model during training. Adam optimizer is used for proper momentum estimation of all the parameters. The learning rate considered for the experimental setup is 0.001. Authors have considered histogram equalization and contrast stretching as discussed earlier in Section 3.1.3 for augmentation purpose. The total training time across complete experiment amounts to 16 hours. Here, training time for individual models for RegNet X and RegNet Y is 3 hours each whereas Swin B Transformer, and VGG-11 took 4 hours each. This variation in training times of individual base model reflects the disparity in model complexity. To optimize memory usage on the 4GB GPU, batch sizes are adjusted dynamically, and mixed-precision training is employed when feasible. Despite hardware constraints, the setup demonstrated adequate capability to handle preprocessing, augmentation, and inference tasks, leveraging frameworks such as PyTorch with CUDA acceleration. This configuration allowed for deploying such models on mid-tier hardware for reproducible research.

As mentioned earlier, all considered base models are pre-trained on the IMAGENET1K V1 dataset, enabling them to leverage learned features from a vast array of images. This pretraining enhances their initial performance on the specific dataset of histopathological images, allowing for more effective transfer learning. By employing this ensemble of diverse CNN architectures, overall predictive accuracy for identifying colon cancer is enhanced.

### 4.2 Dataset

As discussed earlier, the considered LC25000 dataset (a publicly accessible histopathological image repository available at https://academictorrents.com/details/7a638ed187a6180fd6e464b3666a6ea0499af4af) consists of 25,000 high-resolution hematoxylin and eosin (H&E)-stained images evenly distributed across 5 different classes. The proposed ensemble model is devised initially for colon cancer and hence only 10,000 images (colon cancer images) are considered which are split into training and testing dataset in the ratio of 80:20. The performance of various base models and proposed ensemble model is evaluated using different performance metrics namely accuracy, recall, precision, and F1-score. For detailed understanding of these performance metrics and corresponding mathematical formulations, readers may refer (35).

### 4.3 Evaluation of effectiveness of proposed model

The effectiveness of proposed model is evaluated in various aspects as follows:

#### 4.3.1 Comparative analysis of base CNN models and ensemble model

The proposed ensemble model employs weighted averaging with various weight combinations to classify colon cancer histopathological images into adenocarcinoma (C1) and benign (C2). Performance metrics as illustrated in **Table 2** reveal stark contrasts between base models and ensembles:

**Table 2:** Comparative analysis of various models with the proposed ensemble for colon cancer.

Model	Accuracy		Recall				Precision				F1 Score			
	Train	Test	Train		Test		Train		Test		Train		Test	
			C1	C2	C1	C2	C1	C2	C1	C2	C1	C2	C1	C2
RegNet X	0.53	0.52	0.56	0.50	0.56	0.48	0.53	0.53	0.52	0.52	0.54	0.52	0.54	0.50
RegNet Y	0.52	0.50	0.54	0.49	0.53	0.46	0.51	0.52	0.50	0.49	0.53	0.50	0.51	0.48
Swin B	0.99	0.98	0.97	0.99	0.99	0.98	0.99	0.98	0.98	0.99	0.98	0.98	0.99	0.98
VGG 11	0.55	0.53	0.19	0.91	0.19	0.87	0.68	0.53	0.59	0.52	0.30	0.67	0.29	0.65
E1	0.86	0.86	0.74	0.97	0.77	0.95	0.96	0.79	0.94	0.81	0.84	0.87	0.85	0.87
E2	0.95	0.95	0.97	0.98	0.98	0.97	0.98	0.99	0.96	0.98	0.98	0.99	0.97	0.97
E3	0.97	0.98	0.98	0.98	0.99	0.99	0.99	0.98	0.97	0.99	0.99	0.98	0.98	0.99
E4	0.98	0.99	0.99	0.98	1.00	0.99	1.00	0.99	0.97	1.00	0.99	0.98	0.98	0.99

E, Ensemble model (RegNet X, RegNet Y, Swin B, VGG 11).

E1, Weight distribution (0.25, 0.25, 0.25, 0.25).

E2, Weight distribution (0.2, 0.2, 0.4, 0.2).

E3, Weight distribution (0.13, 0.13, 0.6, 0.13).

E4, Weight distribution (0.1, 0.1, 0.7, 0.1).

##### 4.3.1.1 Base model performance

RegNet X/Y underperform significantly (50-52% test accuracy), indicating poor generalization. Swin B achieves 98% test accuracy but exhibits overfitting (99% training accuracy), likely due to its hierarchical architecture’s ability to capture fine pathological details. VGG11 shows moderate performance (53% test accuracy).

**Figure 9:**
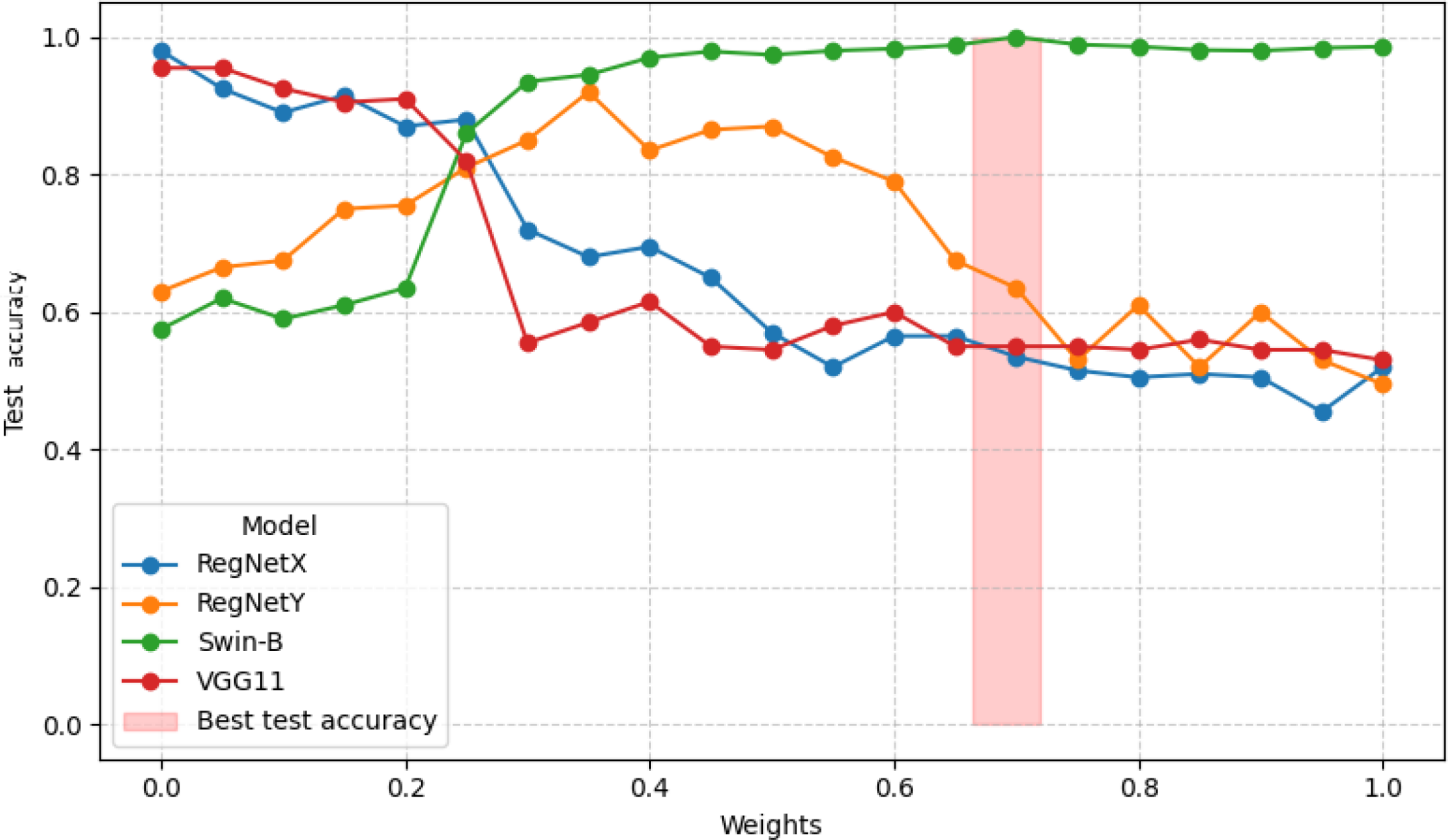
Comparison of test accuracy with different weights across models.

The ensuing test accuracy curves for RegNetX, RegNetY, Swin-B, and VGG11 as illustrated in **Figure 9**, clearly indicate that highest test accuracy is achieved for maximum weightage of Swin-B. Owing to the outperformance of Swin-B model, authors perform a grid search analysis to determine weights of individual base models toward final ensemble model by varyingly increasing Swin-B model's contribution in the range of 0 to 1. It is followed by distributing the remaining weight equally across other three base models. This grid search determines that ensemble setting [0.1, 0.1, 0.7, 0.1] for RegNetX, RegNetY, Swin-B, and VGG11 respectively is the best option, achieving maximum test accuracy and recall across different classes. This evidence advocates the effectiveness of giving a greater weighting to Swin-B in the final ensemble, taking maximum advantage of its discriminative ability while sustaining model diversity for robustness.

##### 4.3.1.2 Ensemble models performance

**Figure 10:**
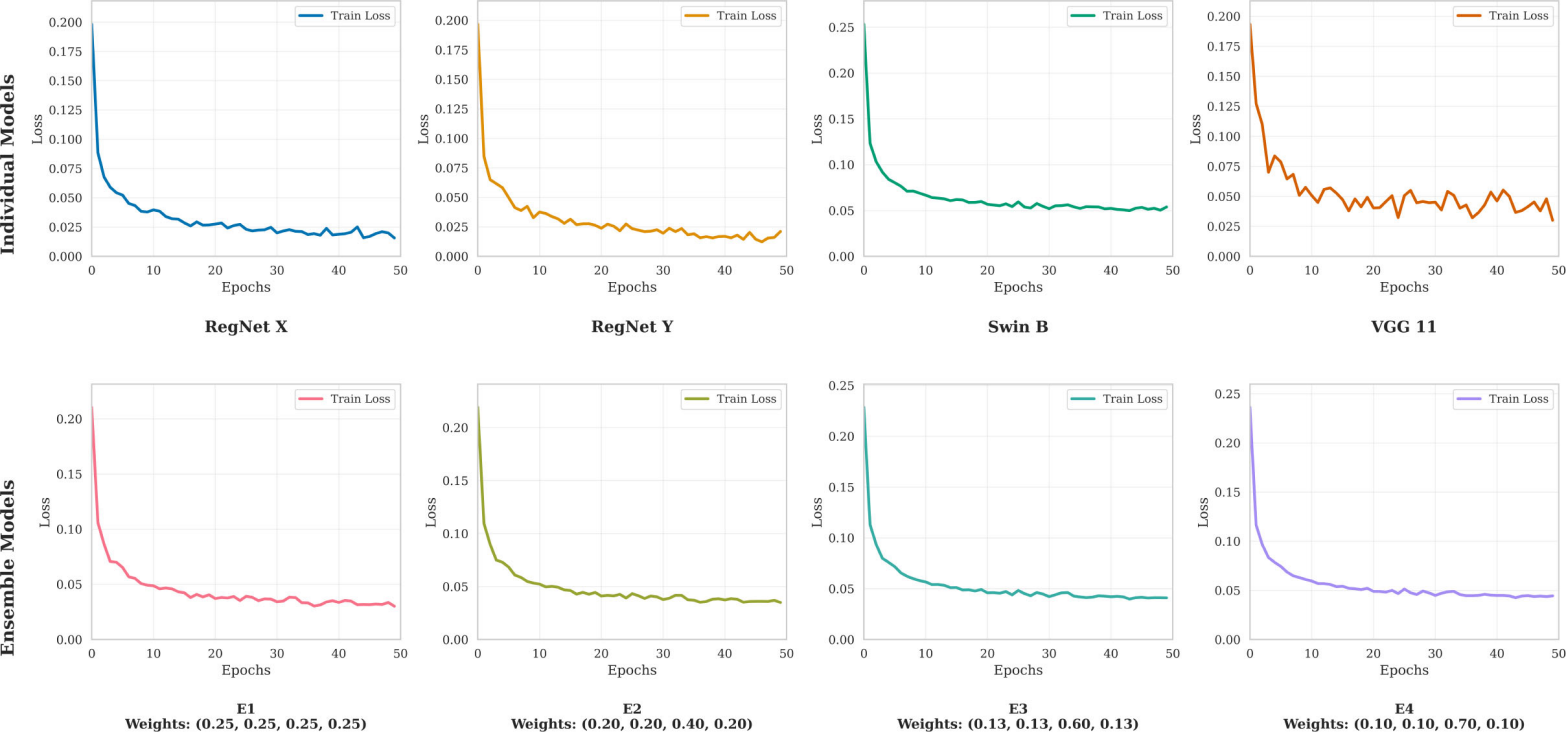
Loss curve plot of individual and ensemble models.

In this experiment, four ensemble configurations (E1–E4) are evaluated using different weight assignments for the base models. As discussed earlier, the weight of Swin B is varied increasingly, while the remaining weight is equally distributed among the other three base models. For example, E1 assigns equal weights of 0.25 to RegNet X, RegNet Y, Swin B, and VGG11. Similarly, E2, E3, and E4 correspond to weight configurations of (0.2, 0.2, 0.4, 0.2), (0.13, 0.13, 0.6, 0.13), and (0.1, 0.1, 0.7, 0.1), respectively. Among the four test cases, E4 emerges as the most robust configuration by assigning 70% weight to Swin B while marginalizing comparatively weaker models. This configuration achieves high test accuracy (99.2%) with reduced overfitting, as reflected by a training accuracy of 98%. The proposed ensemble also achieves high test recall values (99-100%), indicating its strong capability to correctly identify cancer-positive cases while minimizing false negatives. Furthermore, the ensemble diversity helps to mitigate the overfitting tendency of Swin B when used independently. The loss curves of the individual and ensemble models are illustrated in **Figure 10**.

##### 4.3.1.3 Evaluation of ensemble and Swin B model

This subsection presents a comparative analysis of the proposed ensemble model against the primary baseline, Swin B, using both parametric and non-parametric statistical tests conducted over ten randomized experimental runs. For each evaluation metric (Test Accuracy, Recall, and F1-score), the mean and standard deviation are computed across all runs to assess the stability and consistency of the proposed ensemble model.

**Figure 11:**
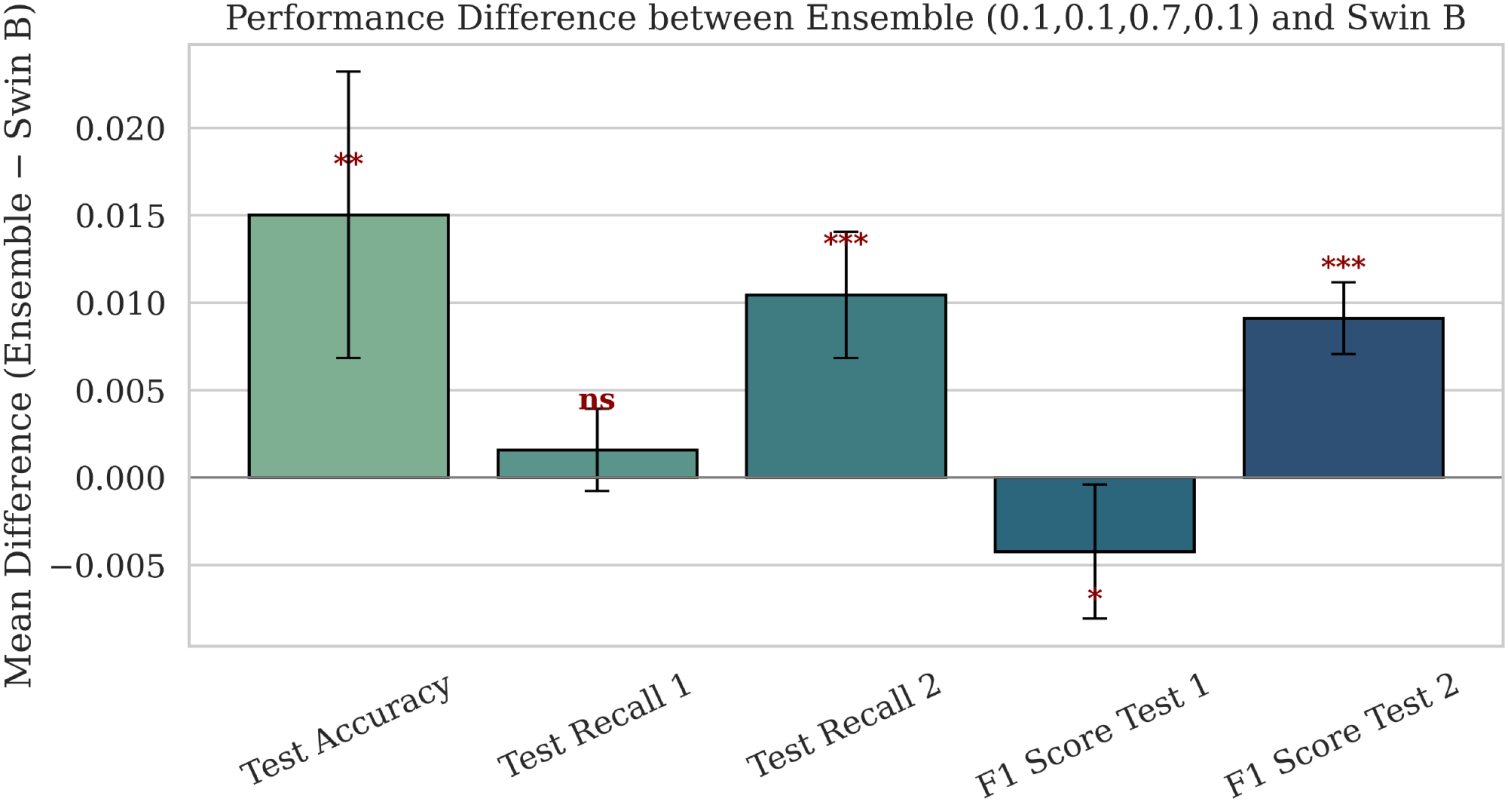
Performance difference between ensemble model and Swin B.

To further assess statistical significance, paired t-tests and Wilcoxon signed-rank tests were conducted to compare the ensemble model with Swin B on all principal metrics. The results, presented in **Figure 11**, indicate statistically significant gains in several metrics, identified by p-value annotations (*, **, ***). Here, *,**, and *** indicate the p-value < 0.1, p-value < 0.05, and p-value < 0.01 respectively which are used to compare ensemble model and Swin B transformer. Specifically, statistically significant positive differences appear in Test Accuracy, Test Recall 2, and F1 Score Test 2, whereas Test Recall 1 presents nonsignificant (“ns”) results, which points to comparable performance between the two models under the given evaluation setting. In summary, the proposed ensemble model has shown consistent and reproducible performance gains over Swin B, both from mean–standard deviation analysis and under rigorous statistical testing.

##### 4.3.1.4 Visualization of images using Gradcam

**Figure 12:**
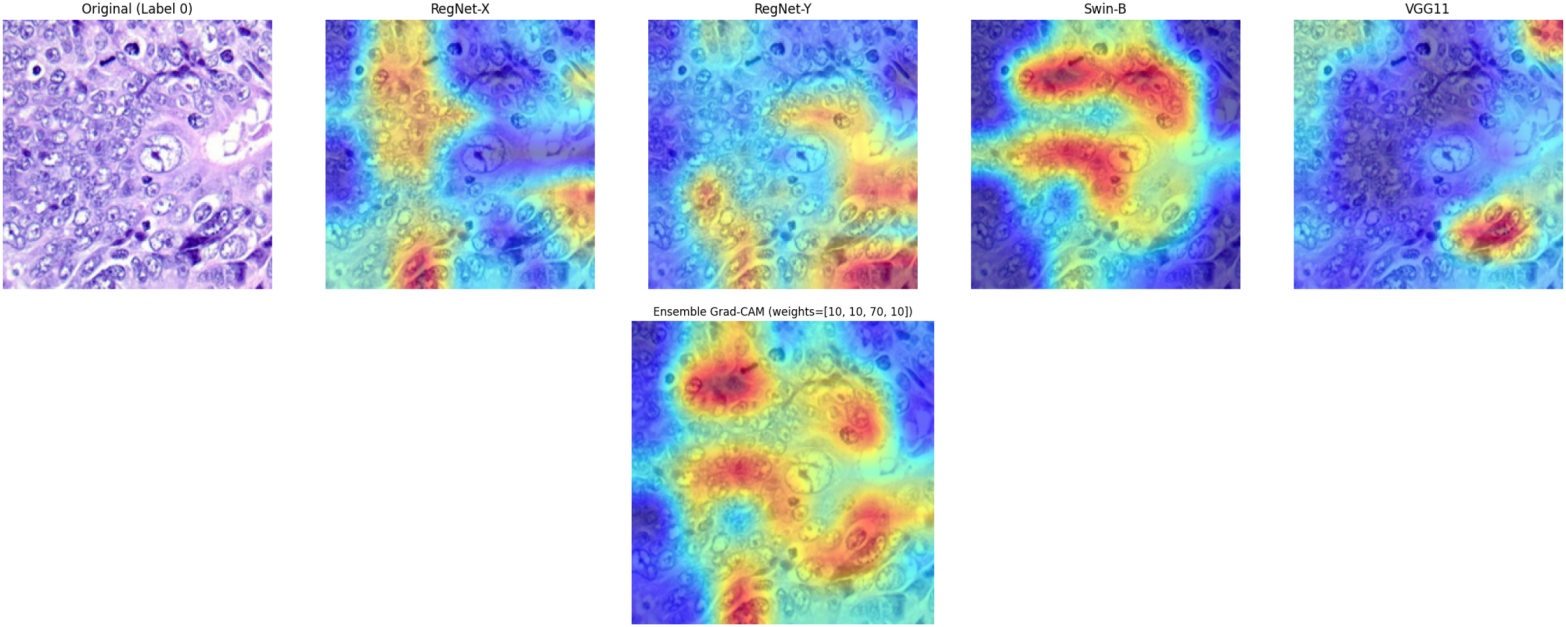
Gradcam visualization of models on cancerous tissue.

To further interpret and visualize the decision making process of deep learning models, authors employed Gradient-weighted Class Activation Mapping (Grad-CAM). Grad-CAM leverages the gradients of any target class flowing into the final convolutional layer to generate a coarse localization map highlighting important regions in the input image, crucial for explaining and validating model behavior, particularly in medical image analysis where interpretability is significant. The Grad-CAM visualizations of cancerous colon tissue for each base model—RegNet X, RegNet Y, Swin B, and VGG11, and proposed ensemble model is illustrated in **Figure 12**. In **Figure 12**, highlighted regions represent areas that contributed most to the classification decision.

##### 4.3.1.5 Key findings

Recall Prioritization: As illustrated in **Table 2**, ensembles E2–E4 achieve superior recall (0.97–0.98 vs. ≤0.95 in base models), critical for minimizing false negatives in healthcare.

Overfitting Mitigation: The ensemble’s aggregated predictions stabilize outputs, addressing Swin B’s overfitting vulnerability.

Generalization: Combining models with varying inductive biases (e.g., Swin B’s attention to fine details + VGG11’s broader feature extraction) ensures robustness against staining variability or scanner artifacts.

This approach demonstrates that no single model suffices for histopathological analysis—ensembles adds up the complementary strengths, delivering consistent, generalizable performance essential for clinical deployment.

#### 4.3.2 Evaluating the impact of histopathological image preprocessing

This subsection aims to evaluate the impact of histogram equalization and contrast stretching during histopathological image preprocessing. As discussed earlier, these preprocessing techniques enhance the visibility of subtle details and enable enhanced feature extraction by redistributing pixel intensities, making important features more distinguishable. In order to validate the effectiveness of preprocessing techniques, authors carried out the experiment without preprocessing techniques. The comparative analysis of the model with and without preprocessing can be inferred from **Table 3**. It presents the differences (delta values) between both cases. Here class L1 and L2 represents Colon adenocarcinoma and Colon benign tissue respectively.

**Table 3:** Delta analysis of various models with and without image preprocessing.

Model	Accuracy		Recall				Precision			
	Train	Test	Train		Test		Train		Test	
			L1	L2	L1	L2	L1	L2	L1	L2
RegNet X	0.020	0.020	0.040	0.030	0.040	-0.020	-0.0117	0.0519	-0.0215	0.0247
RegNet Y	0.020	0.012	0.110	0.030	0.006	0.030	0.0142	-0.0343	-0.0297	-0.0634
Swin B	0.150	0.150	0.103	0.080	0.230	0.090	0.1210	0.0600	0.1323	0.0600
VGG 11	0.100	0.040	0.069	0.094	0.050	0.092	0.1085	0.0290	0.0938	0.0519
E1	0.240	0.205	0.126	0.159	0.127	0.165	0.0810	0.1126	0.0970	0.2261
E2	0.200	0.180	0.181	0.100	0.200	0.156	0.1100	0.1201	0.1140	0.0850
E3	0.190	0.224	0.230	0.100	0.135	0.140	0.1130	0.1164	0.0800	0.0801
E4	0.111	0.110	0.160	0.070	0.110	0.080	0.1000	0.0860	0.0320	0.0420

E, Ensemble model (RegNet X, RegNet Y, Swin B, VGG 11).

E1, Weight distribution (0.25, 0.25, 0.25, 0.25).

E2, Weight distribution (0.2, 0.2, 0.4, 0.2).

E3, Weight distribution (0.13, 0.13, 0.6, 0.13).

E4, Weight distribution (0.1, 0.1, 0.7, 0.1).

The comparative analysis illustrated in **Tables 2**, **3** clearly establishes the effectiveness of histogram equalization and contrast stretching in terms of accuracy, recall, and precision. Thus, it is evident that considered image preprocessing techniques helps in enhancing the effectiveness of proposed model.

This outperformance of proposed model is achieved as a result of using an ensemble model in collaboration with preprocessing techniques. While ensemble model harnesses the strength of each base model, bias of individual model is reduced due to combination of multiple models. Further, ensemble model aims to achieve a robust model enabling to obtain a generalized model. Additionally, this outperformance may also be attributed to preprocessing techniques such as histogram equalization and contrast stretching, aiming toward enhancing the feature visibility.

#### 4.3.3 Ability to achieve a generalized cancer detection ensemble model

As discussed earlier, the proposed model (which is trained for colon cancer images) is also tested for lung cancer images in order to see if it generalizes well. In the considered dataset, 15,000 images are present for 3 different classes pertaining to lung cancer as illustrated in **Figure 2**. Here, lung malignancies represent similarities between lung carcinoma and colon carcinoma. Ahead of testing the model for lung cancer, histogram equalization and contrast stretching are carried out to enhance tissue clarity and leveraging the ensemble’s ability. The obtained results are illustrated in **Table 4**. For lung cancer predictions, there were no signs of data leakage, and the model performed stably without any additional training. Here, the model outputs colon carcinoma for tissue associated with lung carcinoma and colon benign tissue for images associated with lung benign tissue. In **Table 4**, C1 represents lung carcinoma and C2 represents lung benign tissue. Hence, it is evident from results illustrated in **Table 4** that the model has successfully generalized over the homogenuity of colon tissue and lung tissue.

**Table 4:** Comparative analysis of various models with proposed model for lung cancer.

Model	Test accuracy	Recall		Precision	
		C1	C2	C1	C2
RegNet X	0.4963	0.5381	0.4995	0.4996	0.4914
RegNet Y	0.4933	0.5298	0.4487	0.4601	0.4826
Swin B	0.9822	0.882	0.9796	0.9807	0.9892
VGG 11	0.6036	0.22	0.8846	0.5794	0.5031
E1	0.655	0.951	0.7336	0.8371	0.9371
E2	0.950	0.974	0.829	0.792	0.9882
E3	0.985	0.944	0.9728	0.973	0.9921
E4	0.990	0.980	0.9921	0.980	0.9963

The ensemble model demonstrates robust recall and precision metrics in distinguishing critical tissue subtypes, such as lung adenocarcinoma (C1) from lung benign tissue (C2) —a clinically vital differentiation for guiding personalized therapies. We obtained encouraging results, suggesting that the framework can be generalized, capable of enhancing consistency in cancer detection.

While the results are promising, there are two major limitations:

1. Dataset Constraints: Training on 25,000 histopathological images—limited to colon and lung tissues—restricts the model’s exposure to broader histological diversity. Furthermore, the evaluation was carried out only on images from LC2500 dataset. Future work should expand testing to include rare cancer subtypes and multi-organ datasets to validate generalizability.
2. Computational Burden: The base models’ large parameter counts (e.g., Swin B: 87.8M, VGG11:138.4M) prolong training times and demand high-end hardware, hindering scalability for resource-constrained clinics. Optimizing model architectures or exploring lightweight variants could address this bottleneck.

Despite these challenges, the framework’s ability to harmonize accuracy, recall, and generalizability establishes a foundation for advancing automated cancer diagnostics. Addressing these limitations in future iterations could further bridge the gap between research innovation and clinical utility.

## 5 Conclusion and future directions

This study highlights the effectiveness of an ensemble approach that integrates four distinct CNN architectures—RegNet X, RegNet Y, Swin B, and VGG11—for the classification of histopathological images, with a particular emphasis on colon cancer. By combining the unique strengths of each model and offsetting their individual limitations, the ensemble enhances both classification accuracy and robustness. The integration of advanced image preprocessing techniques, such as histogram equalization and contrast stretching, further refines the input data, contributing to improved performance.

The ensemble consistently outperforms individual models, demonstrating strong potential for reliable cancer detection. The consistently high recall that the proposed ensemble demonstrates confirms the effectiveness and supports its suitability for sensitive histopathological classification contexts. This adaptability underscores the model’s relevance for multi-cancer diagnostic applications.

While the results are encouraging, the study does face challenges related to data limitations and computational complexity. Future efforts will aim to broaden the dataset and explore hybrid or lightweight model architectures that maintain performance while reducing resource demands.

In conclusion, the proposed ensemble framework marks a meaningful step forward in automated histopathological analysis. Its robustness, accuracy, and adaptability position it as a promising tool for enhancing diagnostic workflows, with the potential to support timely and dependable cancer detection across diverse clinical contexts.

## Data Availability

The original contributions presented in the study are included in the article/supplementary material. Further inquiries can be directed to the corresponding author.
